# Getting to the Heart of Left–Right Asymmetry: Contributions from the Zebrafish Model

**DOI:** 10.3390/jcdd8060064

**Published:** 2021-06-04

**Authors:** Kelly A. Smith, Veronica Uribe

**Affiliations:** Department of Physiology, The University of Melbourne, Parkville, VIC 3010, Australia; veronica.uribesokolov@unimelb.edu.au

**Keywords:** left–right patterning, asymmetry, laterality, heart development, zebrafish

## Abstract

The heart is laterally asymmetric. Not only is it positioned on the left side of the body but the organ itself is asymmetric. This patterning occurs across scales: at the organism level, through left–right axis patterning; at the organ level, where the heart itself exhibits left–right asymmetry; at the cellular level, where gene expression, deposition of matrix and proteins and cell behaviour are asymmetric; and at the molecular level, with chirality of molecules. Defective left–right patterning has dire consequences on multiple organs; however, mortality and morbidity arising from disrupted laterality is usually attributed to complex cardiac defects, bringing into focus the particulars of left–right patterning of the heart. Laterality defects impact how the heart integrates and connects with neighbouring organs, but the anatomy of the heart is also affected because of its asymmetry. Genetic studies have demonstrated that cardiac asymmetry is influenced by left–right axis patterning and yet the heart also possesses intrinsic laterality, reinforcing the patterning of this organ. These inputs into cardiac patterning are established at the very onset of left–right patterning (formation of the left–right organiser) and continue through propagation of left–right signals across animal axes, asymmetric differentiation of the cardiac fields, lateralised tube formation and asymmetric looping morphogenesis. In this review, we will discuss how left–right asymmetry is established and how that influences subsequent asymmetric development of the early embryonic heart. In keeping with the theme of this issue, we will focus on advancements made through studies using the zebrafish model and describe how its use has contributed considerable knowledge to our understanding of the patterning of the heart.

## 1. Introduction

Whilst vertebrates are seemingly symmetrical externally, there is considerable asymmetry when it comes to internal organs and viscera. Heart, gut, liver, stomach and spleen are all asymmetrically patterned; at the organ level, in their position within the body cavity and relative to one another. The correct asymmetric positioning of organs (termed situs solitus) is required for the correct alignment and connection of organs with one another. When laterality is disturbed, pathological consequences ensue [1]. Situs inversus is a condition involving reversal of the internal organs. Whilst the rhetoric is that a complete mirror-image reversal poses no threat to health, individuals with such a reversal are at a significantly higher risk of congenital defects than those with situs solitus [2]. This is a testament to the importance of establishing correct directionality as well as left–right asymmetry. A more severe laterality condition is that of situs ambiguous, which can consist of either left or right isomerisms (i.e., duplicate left or right sidedness) and may be complete or partial [3]. This can result in either an absence or multiplication of organs (such as asplenia or polysplenia, for example), as well as malrotation of major organs and poor connections between organs [3]. 

The heart is particularly sensitive to disturbances in left–right patterning and the most severe symptoms of laterality disorders are related to defects in cardiac patterning [3]. The reason for this is perhaps two-fold: firstly, the heart plays such a pivotal role in our survival that anything affecting its function has dire consequences [4] and, secondly, the organ itself exhibits considerable asymmetry and is, therefore, highly sensitive to left–right patterning defects [5]. A range of laterality defects can affect the heart, and these include AV valve defects, atrial septal defects, ventricular septal defects, transposition of the great arteries, double-outlet right ventricle and outflow tract defects [6]. Understanding the basis of where and when left–right patterning impacts heart development is therefore an important endeavour and may assist in our interpretation of diseases arising from them, as well as diagnosing susceptible individuals.

In zebrafish, the heart exhibits laterality throughout almost all stages of development—from initial formation of the heart fields to tube formation and dextral looping [7]. Both the anatomy and molecular regulation of these events are comparable across vertebrate species [8], making the zebrafish an attractive model to study this process. The genetic tractability as well as embryo accessibility are features that have been exploited for the study of left–right patterning in the heart. Here, we will discuss the contribution this model has made to our understanding of left–right patterning and its impact on heart morphogenesis.

## 2. Establishment of the Left–Right Axis around the Kupffer’s Vesicle

Across species, left–right patterning is controlled by the left–right organiser (LRO): a transient organ positioned caudal to the notochord and emerging toward the end of gastrulation [9]. Known as the Kupffer’s vesicle (KV) in zebrafish (the node in the mouse and gastroceol roof plate in *Xenopus*), in zebrafish it is a spherical and hollow structure lined with motile cilia that generate leftward fluid flow [10,11,12]. Debate exists about how left–right symmetry is initially broken within the embryo, with experiments as far back as the 19th century hinting that it could be as early as the initial two-cell cleavage event [13]. The complexities surrounding this have been reviewed in depth elsewhere [14]. What is not disputed is that left–right asymmetric gene expression is established around the KV and this precedes the amplification of left–right signalling that will be propagated throughout the embryo.

**Figure 1:**
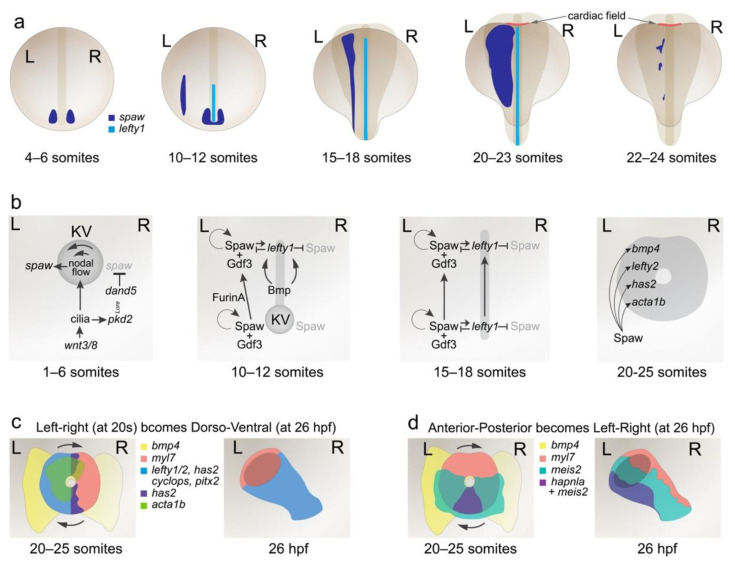
Left–right patterning in the zebrafish axis and cardiac disc. (**a**) Time-course of *spaw* and *lefty1* expression during establishment and propagation of lateral *spaw* expression. *spaw* is initiated peri-KV at 4–6 somites (4–6 s) and becomes enriched on the left side of the node, and expression is induced in the PLPM by 10–12 s. *lefty1* is induced by *spaw* in the midline and expression propagates up the embryo rostrally from 12 to 18 s. At 20–23 s, *spaw* expression is broad and by 24 s, expression is almost extinguished. (**b**) Models of the genetic pathways involved in left–right axis patterning at different stages of zebrafish development. (**c**) At the cardiac disc stage (20–25 s), there is significant left–right patterning within and around the cardiac disc. Upon tube formation, this left–right organisation is repositioned to become dorso–ventral within the tube by 26 h post fertilization (hpf). (**d**) Anterior–posterior markers within the cardiac disc at 20–25 s become repositioned in the cardiac tube to take on left–right asymmetry in the heart tube at 26 hpf. A more detailed representation of cardiac morphogenesis is depicted in Figure 2.

One of the earliest reported asymmetrically expressed genes around the KV is that of *southpaw*/*spaw*, a TGF-beta family ligand and instructive regulator of left–right patterning. *spaw* (known as Nodal in the mouse) is expressed around the KV from 4 to 6 somites (4–6 s) in a symmetrical manner [15] and functionally requires heterodimerisation with Vg1/Gdf3, also a TGF-beta family ligand [16] (Figure 1). At 10–12 s, expression becomes asymmetrically expanded on the left side and is simultaneously induced in the left side of the lateral plate mesoderm (LPM) [15] (Figure 1).

The initial asymmetric expression of *spaw* around the KV is dependent on leftward nodal flow within the KV. Embryos with defective KV or cilia formation, such as *no tail*/*ntl-* and *polaris*-deficient animals, present with organ laterality defects and this has been traced to mispatterned *spaw* expression [10,17]. Indeed, genes, such as *shp2*, that were associated with cardiac morphogenesis, have been discovered, upon closer examination, to play a role in cilia formation of the KV, rather than in the heart itself [18]. Leftward nodal flow contributes to asymmetric *spaw* expression on the left side of the KV and it also regulates asymmetric right-sided expression of the *spaw* antagonist, Cerl2/Dand5 [19]. Dand5 is a secreted Cerberus/Dan family ligand and expressed earlier than *spaw* at 2–3 s around the KV [20]. Interestingly, its expression is symmetrical until 8–10 s, at which time its expression becomes higher on the right side of the KV [20]. This asymmetry in *dand5* expression immediately precedes the asymmetric shift in *spaw* expression (by 1 h), indicating the tight temporal regulation of this process. Loss-of-function for *dand5* results in bilateral *spaw* expression around the KV and in the LPM [21] (Figure 1).

Upstream of *dand5* is the *curly up* (*cup*)/*pkd2* gene, which encodes a Ca^2+^-activated non-specific cation channel, Polycystin 2 (Figure 1). Zebrafish *cup* mutants show defects in asymmetrical positioning of several organs, including the heart. In *cup* mutants, the expression of *spaw* becomes bilateral [22]. The *pkd2* gene is necessary for the formation of the intraciliary calcium oscillations on the left side of the KV at 1–4 s and those oscillations are required upstream of the asymmetric expression of *dand5* and *spaw* [23].

Peri-KV *spaw* expression is also reduced upon downregulation of *wnt3* and *wnt8*, and this leads to absent or randomized expression of *spaw* in the LPM [24]. Similarly, temporal blockage of the Wnt pathway through heat-shock induction of the pathway inhibitor, Dkk1, at 3 s also alters *spaw* expression [25]. Interestingly, these Wnt ligands (Wnt3 and 8) play a role both in the earliest steps of left–right patterning, by controlling ciliogenesis at the KV [25] (Figure 1), but also at later stages, through Gata4, where they control the competence of the heart field to respond to asymmetric cues [24].

In the mouse, Spaw/Nodal is described to induce its own expression [26] and this is a key feature of Spaw activity because, once asymmetrically expressed, it is enhanced, reinforcing asymmetric patterning (Figure 1). Several lines of evidence suggest that this autoregulation is responsible for its own induction in the posterior LPM (PLPM), although the precise mode of how this is achieved remains to be demonstrated. What is known is that loss-of-function mutants for *spaw* show gene expression around the KV, but never in the LPM [21]. Consistent with this, mutants with defective processing and extracellular release of Spaw also fail to induce *spaw* in the PLPM [27]. Conversely, overexpression of the proprotein convertase, FurinA (which catalyses synthesis of mature Spaw), results in faster induction of *spaw* in the anterior LPM (ALPM) [27], adding further evidence to autoregulatory activity of Spaw. Spaw activity is also amplified by heterodimerisation with Gdf3 (Figure 1). This heterodimerisation increases the long-range action of Spaw ([28]; initially demonstrated for Gdf1 in the mouse [29]) and Gdf3 loss-of-function (LOF) results in an absence of *spaw* expression in the LPM [28]. Finally, direct injection of Nodal protein or mosaic expression of *spaw* in the ALPM is sufficient to induce *spaw* expression [27,28]. Together, these data describe that Spaw, Gdf3 and FurinA are essential for Spaw induction in the LPM and this may be directly through Spaw/Gdf3 activity. Ultimately, misexpression of *spaw*, either through loss-of-function or bilateral expression in the LPM, is associated with incorrect organ asymmetry, including of the heart, which is a testament to the instructive nature of Spaw in the process of left–right patterning.

## 3. Propagation of Left–Right Signalling from Posterior to Anterior LPM

Once expression of *spaw* begins in the PLPM, it propagates from posterior-to-anterior up the left side of the embryo (Figure 1). Multiple factors exist to both maintain *spaw* expression in the left LPM and to restrict it from the right. Anatomically, the embryonic midline is recognised as essential for left-sided *spaw* expression. Early studies identified several mutants (including *ntl*, *floating head* and *cyclops*) with floorplate or midline defects that associate with laterality defects of the heart [30], suggestive of a “midline barrier”. It has since become appreciated that the ligand, Lefty1, is expressed in the midline [31] and is a major determinant in maintaining left-sided *spaw* expression (Figure 1).

Like Spaw, Lefty1 is a TGF-beta family ligand, and has a complex relationship with Spaw: Lefty1 inhibits *spaw* expression, whereas Spaw induces *lefty1* expression (Figure 1). This has been substantiated with *lefty1* LOF models that show bilateral *spaw* in the LPM [32,33] and *lefty1* overexpression resulting in an absence of *spaw* expression [34]. By contrast, *spaw* LOF results in an absence of *lefty1* expression [15,21], supporting a role for Spaw in inducing *lefty1*. The result of this regulatory loop has been elegantly demonstrated with the dynamics of gene expression for these two genes: *spaw* expression propagates up the LPM immediately ahead of *lefty1* expression [21,35] and inhibition of *lefty1* results in a faster propagation rate for *spaw* [21]. Notably, in instances when *lefty1* expression is absent, a local supply of Nodal protein is sufficient to induce *lefty1* [36], validating this regulatory loop. One confounding factor is how this co-regulation can result in expression of *spaw* at all. Spaw can autoregulate itself and is highly expressed on the left side. It is plausible that a threshold level of *spaw* expression exists to overcome *lefty1* inhibition on the left side but not on the right, explaining the maintained asymmetry. Data from different biological contexts exists to support this idea: treatment with a Nodal inhibitor can overcome embryonic defects caused by *lefty* LOF [32]. Conversely, co-expression of *lefty* with *nodal* restores phenotypes observed from overexpression of *nodal* alone [34]. These suggest that a strict balance of these two ligands must be in place and that a major role for Lefty proteins is to dampen Nodal activity.

Bmp signalling has also been demonstrated to be important in maintaining *spaw* expression on the left side and its activity functions through *lefty1* [36] (Figure 1). Loss of Bmp activity by either ligand or receptor LOF models results in bilateral *spaw* expression and left–right patterning defects of the heart [37]. This was shown to coincide with reduced *lefty1* expression. Reciprocally, overexpression of Bmp signalling inhibited *spaw* expression in the LPM and induced *lefty1* in the midline [36,37]. Importantly, overexpression of Bmp signalling was incapable of inhibiting *spaw* expression upon *lefty1* knockdown, demonstrating that the effect of Bmp signalling on *spaw* was through *lefty1* [36].

Together, this describes several aspects of how the identity of the left side is established in the embryo and implies that the right side is formed by default. There have been some reports that signalling exists to also exert right-sidedness; however, this has been a subject of debate and additional evidence to support it remains to be presented [38,39].

## 4. Differentiation of Cardiac Progenitors and Cardiac Fusion

**Figure 2:**
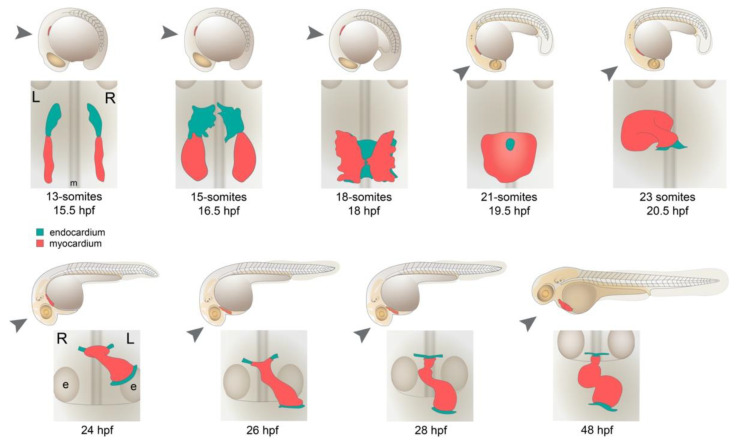
Heart morphogenesis in the zebrafish embryo. Embryonic stages depicted above, with view of heart fields underneath (point of view for heart fields depicted with grey arrow). Endocardial, followed by myocardial, progenitors differentiate in the ALPM by 13 s (15.5 hpf) as bilateral populations. Endocardial cells migrate medially and then caudally to fuse at the midline between the myocardia. The myocardium (comprising cardiomyocytes) then fuses at the midline, encapsulating the endocardium. Between 19 and 26 hpf, the heart undergoes a complex reorganisation, elongating the disc to a tube, where the edges of the cardiac disc reposition under the left eye of the embryo. From 26 hpf, the tube moves back towards the midline and extends anteriorly, positioning the heart over the yolk, between the eyes. The future ventricular chamber realigns to the right and the heart loops, forming an S-shape by 48 hpf. Embryonic midline (m) and eyes (e) depicted to provide anatomical context.

During the establishment of left–right axis patterning in the embryo, formation of the cardiac fields begins. The heart fields emerge as bilateral populations of cells in the ALPM which, by 7 s, express *hand2*, *nkx2.5* or *scl*/*tal1* [40]. The fields do not exhibit obvious asymmetries at this time, and this is consistent with coincident expression of *spaw* confined to the KV region. The early heart consists of endocardial and myocardial progenitors and they are arranged with the endocardium situated anterior to the myocardial fields [41] (Figure 2). By 10 s, endocardial cells differentiate, expressing *fli1a*, [42], followed by myocardial progenitors at 13 s expressing *myl7* (formerly referred to as *cmlc2*; [43]). At these time points, both the endocardium and myocardium are reported to be symmetrically patterned.

At approximately 14 s (16 hpf), endocardial cells migrate in a caudomedial direction, positioning themselves between the bilateral myocardia. From 15 s, the endocardium fuses at the midline [44], followed by medial migration and fusion of the myocardia beginning at 18 s [43] (Figure 2). By 20 s, the myocardium has fused at the midline, forming a disc or cone structure that encapsulates the endocardium [43,44]. At this time, asymmetric expression of *fli1a* and *nkx2.5* has been reported, with expression of both markers extending more posteriorly on the left side [42].

As these differentiated cardiac tissues fuse at the midline, cells that reside more laterally (in the ALPM) remain undifferentiated. Despite this, ALPM will contribute to all regions of the heart, including the ventricle, atrium and inflow and outflow tracts. A lineage tracing experiment using photoconverted Kaede showed that ALPM populations in the 18 s embryo will go on to contribute to the heart by 48 hpf [45]. Interestingly, this contribution is also asymmetric, although it is opposite to what is observed for the medial, differentiated cardiac disc: almost double the number of cells from the right side of the ALPM contribute to the heart than those from the left side. This asymmetry is regulated by *tbx5a,* whereby mutants for *tbx5a* have reduced contribution from the right, comparable to that of the left side [45].

By 20–22 s, several markers exhibit clear left–right asymmetry in and around the cardiac disc (Figure 1). Bmp4 is expressed across the LPM and, by 20 s, becomes enriched on the left side [30]. Two out of three studies examining downstream signalling of Bmp have shown increased activity on the left side of the disc [46,47,48]. Notably, *lefty1*, *lefty2, cyclops* and *pitx2* are expressed exclusively in the left side of the cardiac disc at 22 s [37], and *acta1b* and the ECM biosynthetic enzyme, *has2*, become strongly enriched on the left side of the heart disc by 25 s [46,49]. To date, the only reported factor enriched on the right side is that of phosphorylated myosin light chain II—an upstream regulator of non-muscle myosin II [48]. Anything altering the asymmetric expression of *spaw* (such as *spaw* or *bmp4* knockdown) disrupts the asymmetric patterning of these markers [37,46,48,49], demonstrating that their induction within and surrounding the cardiac field is dependent on left–right axis patterning.

The role of Bmp signalling in this process is complex and complicated by the fact that it plays multiple and diverse roles in left–right patterning of the heart. In addition to Bmp signalling playing an inductive role in *lefty1* expression at early somitogenesis stages, Bmp also signals to the developing cardiac fields. Schilling and colleagues [42] showed that disruption to Bmp signalling (by Bmp4 overexpression) perturbed cardiac laterality but did not affect gut looping, suggesting that these two processes could be decoupled. This observation was later confirmed using temporally inducible transgenics that overexpress either *noggin3* (a Bmp signalling inhibitor) or *bmp2b* [37]. Inhibition of Bmp signalling at the tailbud (late gastrulation) stage resulted in bilateral *spaw* expression across the LPM, a loss of *pitx2* expression, an absence of both *lefty1* and *lefty2* expression and gut and heart laterality defects. When inhibition was performed at 16 s, however, only cardiac laterality was affected [37]. Interestingly, knockdown of *spaw* resulted in symmetrical expression of *bmp4* across the ALPM at 22 s, instead of left side-enriched expression, suggesting that Bmp is acting both upstream of *spaw* (via *lefty1*) as well as downstream. These data demonstrate that not only is left–right patterning of gut and heart temporally separable, but that Bmp signalling is required repeatedly in development, in different contexts for correct left–right patterning of the heart.

## 5. Formation of the Cardiac Tube

At 25 s (21.5 hpf), the heart undergoes substantial reorganisation from a shallow cone or disc-like structure to a linear tube (Figure 2). This process of tube formation, termed “cardiac jogging” [30], relocates the developing heart in an anterior and leftward direction. Indeed, the process of cardiac jogging is the first visible break in symmetry in the embryo from a gross anatomical perspective. At a more detailed tissue level, the initiation of this process has been described as “involution”; the apex of the cardiac cone tilts to the right side of the embryo, creating an involute on the right side of the disc [50], whilst the left side extends and elongates to the left side of the embryo (Figure 2). Over approximately 6 h, the peripheral edges of the disc (which also contribute to the future atrium) will come together to form the inflow tract of the heart, positioned to the anterior and left side of the embryo, whilst the apex anchors the future outflow tract to the midline (Figure 2) [43].

The process of cardiac jogging has been visualised by long-term confocal timelapse imaging and the migration patterns of cardiomyocytes have been examined. An overall clockwise rotation of the disc has been described by multiple groups [46,51,52]. Cardiomyocytes in the posterior region of the disc migrate faster and further than cardiomyocytes in the anterior half of the disc, contributing to a rotation of 30 to 36 degrees, relative to the original orientation of the disc [46,52]. This rotation has an important consequence on the overall organisation of the heart: as the disc involutes to becomes a tube, the left side of the disc becomes the dorsal surface of the tube and the right side, the ventral surface. This rearrangement has been shown by *lefty2* staining [46,50,51] (Figure 1). This also means that the posterior half of the disc becomes the left side of the tube. This has been substantiated by two different groups with two independent markers of the posterior half of the disc, namely *meis2b* and *hapln1a,* both of which come to occupy the left side of the jogged cardiac tube [53,54], imbuing the heart tube with renewed left–right asymmetry (Figure 1).

The molecular regulation of cardiac jogging and cardiomyocyte migration has been studied in some depth and several factors have been shown to be important for it to take place. Spaw is one such factor that contributes to the migration of cardiomyocytes [52]. Analysis of mutants for the Spaw co-receptor, *one-eye pinhead* (*oep*), showed both speed and directionality of cardiomyocytes is reduced and altered, respectively, in mutants. This results in reduced cardiac disc rotation and hearts that jog to the midline [52]. This effect on cardiomyocyte behaviour was attributed to disturbances in Bmp signalling, downstream of Spaw [52].

As described above, the Bmp signalling pathway is required for correct cardiac jogging in a manner that is temporally separable from its role in patterning the gut and viscera [37]. Bmp is instructive of cardiac jogging, whereby a localised source of Bmp protein (via implantation of a Bmp-soaked bead) can direct cardiac jogging towards the Bmp source [46]. This phenomenon is likely due to its effect on cardiomyocyte migration dynamics, which is impacted by either a loss or gain of Bmp signalling; reduced bmp signalling results in cardiomyocytes that appear sluggish in their migration during cardiac jogging, whereas excessive Bmp activity results in cardiomyocytes that migrate in an apparently over-stimulated and directionless fashion [46]. Both contexts have the same outcome: a midline jog phenotype, demonstrating how important the source of Bmp signal is. Together, these data support a role for Bmp as a chemoattractant for cardiomyocyte migration.

The extracellular matrix (ECM) is also important for cardiomyocyte migration and, therefore, cardiac jogging. The ECM functions both as a substrate for cardiomyocytes to migrate across and presumably also impacts on chemokine propagation. In terms of cardiac jogging, this has been demonstrated for the ECM component, hyaluronic acid (HA). As described above, *has2,* a biosynthetic enzyme of HA, is expressed asymmetrically on the left side of the disc immediately prior to cardiac jogging [46] and presumably deposits extracellular HA asymmetrically through its activity. Asymmetric cardiomyocyte migration is dependent on *has2* function, as *has2*-deficient embryos have cardiomyocytes that migrate at slower rates and only in an anterior direction, resulting in heart tubes positioned in the midline. *has2* has been shown to genetically interact with Bmp signalling, whereby the heart tube is unable to respond to a localised source of Bmp protein when *has2* is absent [46]. This suggests that HA is important for the correct propagation of Bmp signal and/or promoting its activity. In conflict with this, a role for *has2* in inhibiting Bmp signalling has been reported [48]. The authors showed that clonally overexpressing *has2* at high levels resulted in a cell autonomous absence or reduction in Bmp activity in *has2*-expressing cells, whilst adjacent neighbouring cells appear responsive to Bmp. Given the non-cell autonomous function of *has2*, these observations are difficult to resolve and warrant further investigation.

## 6. Looping Morphogenesis

Following cardiac jogging, the linear heart tube rotates and bends into an S-shaped loop in a process called cardiac looping (Figure 2). From 26 hpf, the ventricle moves rightwards, back towards the midline. Soon after, development of the atrioventricular canal initiates (AVC; the region of valve formation), forming a constriction between the chambers. This changes the heart from a linear tube into a segmented, multichambered heart, reorienting the cardiac chambers relative to one another. Functionally, this alters the path of blood flow and, importantly, changes contraction from peristaltic to asynchronous beating of the chambers, minimizing backflow and creating more efficient blood propulsion. As the chambers realign, they start to expand through cell shape and cell adhesion changes, creating “outer curvatures” of the chambers in a process termed “ballooning” [55,56]. From 48 hpf, the chambers continue to realign. The atrium moves medially and, from a ventral perspective, repositions itself behind the ventricle by 5 days post fertilisation [57].

Fate-mapping studies following labelled cardiomyocytes during cardiac looping have developed an organ-scale view of some of these rearrangements. At 28–30 hpf, a rotation of the tube has been described, bringing the dorsal surface (originally the left side of the disc) back to the left side of the looping heart [51]. This was substantiated by *meis2b* expression, which labels the posterior half of the disc prior to tube formation, and becomes expressed on the ventral surface of the tube at 30 hpf [53]. Analysis of either *spaw*-deficient embryos or ciliogenesis mutants shows the direction of this is dependent on the direction of jog but does not impact looping directionality [51]. A little later, between 40 to 48 hpf, a twisting or torsion of the tube occurs [58] and multicoloured cell lineage tracing has shown this involves the ventricle and atrium rotating in opposite directions from one another, either side of the AVC [59].

Organ-intrinsic and -extrinsic factors are both involved in looping of the heart and the activity of *spaw* is one such extrinsic factor. In two independent reports, loss of *spaw* signalling was shown to disrupt cardiac jogging and, to a lesser extent, cardiac looping [49,60]. This is an interesting observation; whilst a higher frequency of embryos with disrupted *spaw* disturb cardiac looping laterality, the majority still undergo dextral looping. This demonstrates that looping can be uncoupled from cardiac jogging and that Spaw contributes to cardiac looping. Despite this, cardiac looping still occurs and with an asymmetry that is seemingly intrinsic or independent of left–right axis patterning.

Another extrinsic contribution to cardiac looping is the addition of cells to both poles of the heart from the second heart field (SHF; [61,62]). With these additions, the heart tube grows but its dimensions are restricted within the pericardial cavity. Model simulations suggest that this kind of elongation in a confined space is sufficient to drive cardiac looping, although directionality is random. A subtle displacement of the caudal portion to the left is enough to drive the buckling to form mostly dextral loops [63]. Indeed, zebrafish mutants, where SHF addition is disrupted, show looping defects [62,64,65,66,67]; however, whether the cellular contribution in the wildtype context is asymmetric is yet to be examined.

Another aspect to consider is the contribution of flow. Given that blood flows through the heart during looping, it is reasonable to speculate that flow plays a role in cardiac looping as one of the extrinsic forces. However, experimental data does not support this hypothesis. Firstly, preventing the heart from beating (and, by extension, blood flow) either by chemical or genetic means does not alter formation of an S-shaped heart [68,69]. It does impact chamber ballooning and valve development, both of which contribute to heart morphogenesis and could be described to “amplify” asymmetries in cardiac looping morphology; however, dextral looping is not dependent on either blood flow or heart contraction.

Examples of intrinsic asymmetries in cardiac looping include both gene expression and the distribution of its ECM. Elegant analyses of the ECM residing between the myocardial and endocardial layers (known as cardiac jelly) have shown it to be expanded on the left side of the tube, compared with the right [54]. This expansion was more prominent in the atrium than in the ventricle and coincided with expression of *hyaluronan and proteoglycan link protein 1a* (*hapln1a*). Hapln1a is an ECM component and cross-links HA and proteoglycans, stabilising ECM [70]. Given the asymmetric expression of *has2* in the cardiac disc, this provides further hints that the ECM contributes to cardiac laterality. Somewhat surprisingly, deletion of *hapln1a* had no impact on cardiac jogging; however, it did result in morphogenetic defects of the looped heart. The atrium of the looped heart continued to show asymmetric ECM deposition on the left side by 50 hpf, but this was disrupted in *hapln1a* mutants [54]. The consequence of this was reduced atrial area and curvature, and hearts were overall more elongated.

Perhaps the most definitive experiment to argue that intrinsic factors influence cardiac looping was an explant culture study. By removing hearts at tube stages and culturing them for 24 h, explanted hearts went on to loop, giving rise to S-shaped and mostly dextral-looped hearts [49]. This was a key observation; it demonstrated that without the influence of ongoing extrinsic asymmetric signalling, in the absence of cellular additions or tissue confinement, and without blood flow, the heart goes on to loop asymmetrically. Further analysis using chemical inhibitors showed that inhibition of the acto-myosin network, but not the microtubule network, prevented cardiac looping [49]. Spaw signal can drive asymmetrical *actin1b* expression in the cardiac disc, which may contribute to the later intrinsic nature of this process. Overall, these results suggest that heart looping is mostly governed by heart-intrinsic mechanisms, which are likely established by earlier left–right axis patterning events involving Spaw to increase the robustness of the process.

Apart from these studies mentioned above, surprisingly few mutants have been described with specific cardiac looping defects. Certainly, mutants have been reported with defects in SHF development, with defects in cardiac chamber ballooning or AVC development, and these do impact on cardiac looping; however, genes involved in looping laterality specifically have yet to be described. This suggests that left–right patterning imbues the heart with intrinsic asymmetry by the completion of cardiac jogging and, when combined with SHF additions, chamber ballooning and AVC formation, it is sufficient to complete cardiac looping. An alternative possibility is that anything essential for cardiac looping is also essential for other, earlier embryonic process, precluding analysis of the role in cardiac looping. Future work investigating the molecular regulation of cardiac looping is required to clarify this.

## 7. Future Perspectives

Asymmetries observed in the larval and adult heart continue to be observed for overall organ morphology, chamber alignment, trabeculation pattern and valve formation [57]. Asymmetric gene expression patterns in the adult cardiac chambers are also observed [53,71], suggesting that patterning is maintained and ongoing in the adult heart. To what extent these asymmetries are required for healthy heart function and in what manner they are regulated remains unexplored. One rare example of an adult laterality phenotype that has been investigated is the analysis of surviving adult *spaw* mutants with unlooped hearts [72]. Adults develop disturbed blood flow patterns and exhibit incorrect heart valve remodelling, reminiscent of valve and septal defects observed in patients with laterality defects. The long-term impact of most adult cardiac asymmetry, however, has not been investigated in zebrafish models.

As well as work outlined in this review, there are several studies emerging in online repositories that provide hints of forthcoming areas of research in this field. These include the regulation of asymmetric peri-KV expression [73], control of lefty expression dynamics [74] and mapping of cell behaviours to analyse looping morphogenesis [75]. Whilst much headway has been made in the past two decades, there remains much unknown about the mechanisms of asymmetric heart formation, particularly with regard to the later stages of morphogenesis. These ongoing studies hold importance; it is through this that we improve our knowledge of the fundamentals of biology and, in turn, contribute to our understanding of the timing and mechanism of disease onset.
